# Vaccination coverage and abandonment among children under two years old in Brazil: a time-series study

**DOI:** 10.1590/1984-0462/2024/42/2023116

**Published:** 2024-05-31

**Authors:** Rossana Marchese Bittencourt Enore, Bruna Hinnah Borges Martins de Freitas, Ronaldo Antonio da Silva, Maria Aparecida Munhoz Gaíva

**Affiliations:** a Universidade Federal do Mato Grosso, Cuiabá, MT, Brazil.

**Keywords:** Epidemiological studies, Brazil, Infant, Immunization programs, Vaccination coverage, Estudos epidemiológicos, Brasil, Infantil, Programas de imunização, Cobertura vacinal

## Abstract

**Objective:**

To analyze the vaccination coverage and abandonment rates among children under two years old in Brazil, from 2015 to 2021.

**Methods:**

A time-series ecological study. The dependent variables of the research were "vaccination coverage" and "abandonment rate", both assessed by Brazilian region. The data were extracted in July 2022 from the Information System of the National Immunization Program. The Prais-Winsten technique was used for the trend analysis, with the aid of the STATA 16.0 software.

**Results:**

The mean vaccination coverage in Brazil was 76.96%, with a decreasing trend during the period (Annual Percent Change=-5.12; confidence interval — CI95% -7.81; -2.34); in 2015, the rate was 88.85% and it dropped to 62.35% in 2021. In turn, the overall abandonment rate was 24.00% in 2015 and 9.01% in 2021, with a mean of 10.48% and a stationary trend (Annual Percentage Change=-9.54; CI95% -22.92; 6.12). In 2021, all the vaccines presented coverage values below 74.00% in the country.

**Conclusions:**

The vaccination coverage rate trend among children under two years old was stationary or decreasing for all the immunobiologicals in all Brazilian regions, with the exception of yellow fever in the South and Southeast regions. There was an increase in the abandonment rate trend for the Meningococcal C vaccine in the country and, specifically in relation to the regions, for BCG in the North, Northeast, and Midwest and for Meningococcal C in the North and Northeast.

## RESUMO

**Objetivo:**

Analisar a tendência das taxas de cobertura e abandono vacinal em menores de dois anos no Brasil, no período de 2015 a 2021.

**Métodos:**

Estudo ecológico de séries temporais. As variáveis dependentes da pesquisa foram a cobertura vacinal e a taxa de abandono, avaliadas por região brasileira. Os dados foram extraídos do Sistema de Informação do Programa Nacional de Imunizações em julho de 2022. Para a análise de tendência, empregou-se a técnica de Prais-Winsten com o auxílio do *software* STATA 16.0.

**Resultados:**

Identificou-se que a média de cobertura vacinal no Brasil foi de 76,96%, com tendência decrescente no período (variação percentual anual=-5,12; intervalo de confiança de 95% — IC95% -7,81; -2,34). Em 2015 a taxa foi de 88,85%, caindo para 62,35% em 2021. A taxa geral de abandono, por sua vez, foi de 24,00% em 2015 para 9,01% em 2021, sendo a taxa média de 10,48% e a tendência estacionária (variação percentual anual=-9,54; IC95% -22,92; 6,12). Em 2021, todas as vacinas tiveram cobertura abaixo de 74,00% no país.

**Conclusões:**

A tendência da taxa de cobertura vacinal de crianças menores de dois anos mostrou-se estacionária ou decrescente para todos os imunobiológicos em todas as regiões brasileiras, com exceção da febre amarela nas Regiões Sul e Sudeste. Houve aumento da tendência da taxa de abandono da vacina Meningo C no país e, especificamente em relação às regiões, para BCG no norte, nordeste e centro-oeste e Meningo C no norte e nordeste.

## INTRODUCTION

The World Health Organization (WHO) highlights childhood vaccination as one of the best investments in health, as it is estimated that, thanks to compliance with the basic childhood vaccination scheme, approximately 2.5 million deaths are prevented every year.^1^ It is noteworthy that, since 1975, Brazil has adopted the National Immunization Program (*Programa Nacional de Imunização*— PNI) as a public health strategy with the purpose of coordinating vaccination actions and ensuring the quality of life of the population with prevention of diseases, based on free vaccination of children, adults and aged people.^2^ Currently, the basic vaccination schedule for Brazilian children includes 17 different immunobiologicals, routinely offered in the country's health services.^3^

However, incomplete vaccination schedules can represent a serious public health problem, as it is necessary to achieve high vaccination coverage (VC) to reduce or prevent circulation of a specific infectious agent.^4,5^ Thus, the VC indicators, which show the number of doses administered and the abandonment rate, which estimates the population's adherence when considering the number of individuals that started and did not complete the regime, represent important instruments for monitoring diseases that are of interest for public health.^6,7^

Nonetheless, in recent years, several countries presented reductions in infant VC rates, such as Mexico, Colombia, Paraguay, Venezuela, Jordan and also Brazil, which may directly reflect on the increase in morbidity and mortality, as children's vulnerability is increased, especially in those under two years old.^7-14^ The decrease in vaccination coverage is a process that preceded the COVID-19 pandemic, but has intensified during this period throughout the country. However, it is also influenced by access to vaccination services and by availability of products.^4^

The United Nations Children's Fund (UNICEF) and the WHO point out that disinformation is behind the continuous reduction in childhood vaccinations over the last three decades, accentuated by the COVID-19 pandemic. Thus, the percentage of children in the world that received all three doses of the Diphtheria, Tetanus and Poliomyelitis (DTP) vaccine, for example, dropped from 86.00% in 2019 to 81.00% in 2021. It is noted that the DTP vaccine is used as a key indicator of global immunization coverage.^15^

Despite all the achievements of the Brazilian PNI, several challenges have limited the scope and maintenance of high vaccination coverage, such as fake news, vaccine hesitancy, organization and opening hours of services, fear of adverse events, and efficacy and safety of the vaccines.^5^ In view of this, there is lack of information on the serial vaccination behavior among children under two years of age. All vaccines offered to children have an indication for doses up to two years of age, according to the country's Childhood Vaccination Schedule, and only two of them are indicated at four years of age as a second vaccine booster, namely: the Diphtheria and Tetanus vaccine (dT) and the Poliomyelitis vaccine (PV).^3^

Therefore, this study aimed at analyzing the trend in vaccination coverage and abandonment rates among children under two years of age in Brazil, from 2015 to 2021. These results allow us to know about the situation of childhood immunization and how its indicators have behaved in recent years, especially among infants, a priority group of the Brazilian PNI. It is known that surveilling vaccine coverage and monitoring vaccine abandonment are powerful instruments to target and devise actions aimed at controlling vaccine-preventable diseases in childhood.

## METHOD

This is an ecological study with a time-series design (trend study)^16^ of the vaccination coverage and abandonment rates in Brazil and its regions, between 2015 and 2021. The dependent variables of the research were the following PNI health indicators: VC and abandonment rates,^17^ both assessed by Brazilian region.

The Brazilian territory is politically and administratively organized by the grouping of all 27 Federation Units into five regions: North, Northeast, Midwest, Southeast and South.^18^ The years (from 2015 to 2021) were considered as an independent variable (time). This period of time was chosen because it is not recommended to analyze series with less than seven points, as the trends tend to be non-significant and the small statistical power of the regression analysis tends to make it difficult to identify a significant trend, either increasing or decreasing.^19^

The data were extracted in July 2022 from the Information System of the National Immunization Program (*Sistema de Informação do Programa Nacional de Imunização* — SI-PNI) on the website of the Informatics Department belonging to the Unified Health System (DATASUS) [http://datasus.saude.gov.br/], considering the national calendar recommended for children under two years of age in Brazil (Table 1). Information was obtained about the VC corresponding to each immunobiological agent by region and according to year, as well as about the doses of each vaccine administered by region, according to year.

**Table 1:** Vaccination schedule of children under two years of age according to vaccine, age and recommended scheme, Brazil, 2023.^3^

Vaccine	Recommended scheme		Age and recommended scheme
	Basic scheme	Booster	
Bacillus Calmette and Guérin (BCG)	1 dose	-	At birth (months)
Hepatitis B (HEPB)	4 doses	-	At birth–dose 1 2–dose 2 (PENTA) 4–dose 3 (PENTA) 6–dose 4 (PENTA)
Pentavalent (PENTA)	3 doses	2 doses with the Measles, Mumps and Rubella (MMR) vaccine (DTP)*	2–dose 1 4–dose 2 6–dose 3 15–booster 1 (DPT-R1) 4 years old–booster 2 (DTP-R2)*
Poliomyelitis (POLIO)	3 doses with the Injectable Poliomyelitis Vaccine (IPV)	2 doses with the Oral Poliomyelitis Vaccine (OPV)	2–dose 1 4–dose 2 6–dose 3 15–booster 1 (POLIO-R1) 4 years old–booster 2 (POLIO-R2)*
10-valent pneumococcal (PNEUMO-10)	2 doses	1 dose	2–dose 1 4–dose 2 12–booster (PNEUMO-R1)
Oral Human Rotavirus Vaccine (OHRV)	2 doses	-	2–dose 1 4–dose 2
Conjugate Meningococcal C (MENINGO-C)	2 doses	1 dose	3–dose 1 5–dose 2 12–ooster (MENINGO-R1)
Yellow Fever (YF)	1 dose	1 dose	9–dose 1
Triple viral (SCR)	1 dose	-	12–dose 1 (SCR)
Tetra Viral (SCRV)	1 dose	-	15–dose 1
Hepatitis A (HEPA)	1 dose	-	15–dose 1

* Dose applied after 2 years.

The coverage rates are already provided as calculated by SI-PNI. The formula for calculating coverage is the number of doses applied of the indicated dose (1^st^, 2^nd^ and 3^rd^ dose, single dose or booster dose, according to the recommended schedule for each vaccine) divided by the target population, and multiplied by 100. In the case of the Measles, Mumps and Rubella (MMR) vaccine, as the second dose can be applied with the tetra viral or MMR, the calculation is provided per dose.

The abandonment rates (referring to multidose vaccines in children under one year old) were calculated by dividing the difference between the number of first doses and the number of last doses administered in the vaccination schedule by the total number of first doses, multiplying the result of the division by 100. Low (<5.00%), average (≥5.00% and <10.00%) and high (≥10.00%) abandonment rates were considered.^6^

Prais-Winsten regression was used for the trend analysis of the rates, as described by researchers Antunes and Cardoso,^19^ which foresees first-order autocorrelation correction. The dependent variable was the logarithm of the rates and the independent variable was the years from the historical series. The Annual Percent Change (APC) was calculated by means of the following expression: APC= [– 1 + 10^b^] * 100%. The following was used to calculate the confidence intervals (CIs): 95%CI [-1 +10^b±t*se^.] * 100%, where "*b"* corresponds to the annual increase rate and "*se"* to the standard error, both extracted from the regression analysis, and the *"t"* value is provided by the Student's *t* distribution table. An increasing trend was considered when the rate was positive; decreasing when negative; and stationary when there was no significant difference between its value and zero in the CI.^19^ This analysis was performed with the aid of the STATA 16.0 software.

The study is exempt from submission to a Research Ethics Committee because it evaluates data in the public domain.

## RESULTS

**Figure 1:**
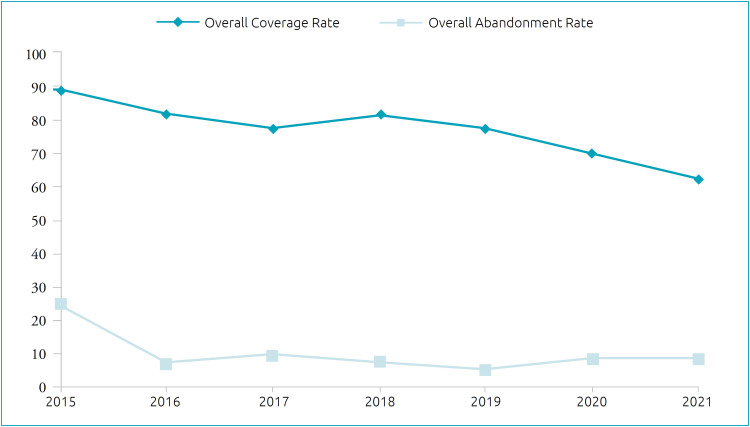
Time series corresponding to the overall coverage and abandonment rate of the immunobiological agents among children under two years old in Brazil (2015–2021).

Between 2015 and 2021, the mean overall coverage rate for the immunobiological agents among children under two years old in Brazil was 76.96%, whereas in 2015 it was 88.85% and, in 2021, 62.35%, with a decreasing trend during the period (APC=-5.12; CI95% -7.81; -2.34). In turn, the mean overall abandonment rate was 10.48%: 24.00% in 2015 and 9.01% in 2021, representing a stationary trend (APC=-9.54; CI95% -22.92; 6.12), as highlighted in Figure 1.

Tables 2 and 3 present the time series corresponding to the VC rate by immunobiological agent (%) among children under two years old in Brazil and by regions, from 2015 to 2021. A decreasing VC trend was observed in Brazil for the following immunobiological agents: BCG, HEPB, PENTA, PNEUMO-10, POLIO, OHRV, MENINGO-C, Triple Viral Dose 1 (SCR-D1), Tetra Viral (SCRV), Pneumococcal Booster 1 (PNEUMO-B1), Meningococcal Booster 1 (MENINGO-B1) and Poliomyelitis Booster 1 (POLIO-B1); and a stationary trend for the others, namely: Yellow Fever (YF), SCR Dose 2 (SCR-D2), HEPA and triple bacterial Booster 1 (DTP-B1). Therefore, no immunobiological agent presented VC values with an increasing trend in the country. An increasing trend in relation to YF was only observed in the Brazilian Southeast and South regions. The other immunobiological agents showed a decreasing or stationary trend in all the Brazilian regions across the years analyzed.

**Table 2:** Time series corresponding to the vaccination coverage rate by immunobiological agent (%) in children under two years old. Brazil, Regions, from 2015 to 2021.

	Region	Rate 2015	Rate 2021	Rate Mean	APC	95%CI	Trend
BCG	North	103.72	74.85	89.76	-4.84	-7.83; -1.75	Decreasing
	Northeast	105.52	66.59	88.75	-7.15	-11.56; -2.51	Decreasing
	Southeast	104.76	65.94	89.23	-7.42	-12.43; -2.12	Decreasing
	South	106.00	74.61	90.91	-4.49	-5.79; -3.18	Decreasing
	Midwest	105.44	74.57	93.78	-5.65	-8.97; -2.21	Decreasing
	Total	105.08	69.01	89.76	-6.54	-10.44; -2.47	Decreasing
HEPB	North	89.43	69.99	80.05	-2.33	-6.57; 2.10	Stationary
	Northeast	90.88	64.09	80.14	-5.09	-10.20; 0.30	Stationary
	Southeast	90.83	56.63	77.39	-8.20	-13.81; -2.23	Decreasing
	South	89.86	61.48	74.67	-4.78	-6.91; -2.60	Decreasing
	Midwest	95.38	69.64	85.86	-5.02	-9.86; 0.07	Stationary
	Total	90.93	61.97	78.79	-6.04	-10.66; -1.19	Decreasing
PENTA	North	85.08	61.31	71.80	-4.83	-6.00; -3.64	Decreasing
	Northeast	95.93	67.69	80.16	-5.70	-7.62; -3.73	Decreasing
	Southeast	99.13	70.91	84.80	-4.90	-6.85; -2.91	Decreasing
	South	98.40	80.03	87.11	-2.95	-4.86; -1.01	Decreasing
	Midwest	95.17	73.65	84.41	-5.11	-7.05; -3.12	Decreasing
	Total	96.30	70.41	82.38	-4.67	-6.08; -3.25	Decreasing
PNEUMO-10	North	75.00	68.11	80.51	-1.88	-7.53; 4.10	Stationary
	Northeast	93.29	70.30	87.21	-4.32	-8.68; 0.26	Stationary
	Southeast	99.01	72.85	89.99	-5.08	-8.26; -1.78	Decreasing
	South	98.44	83.03	92.62	-2.04	-3.03; -1.03	Decreasing
	Midwest	92.76	78.28	91.11	-3.45	-4.81; -2.06	Decreasing
	Total	94.23	73.45	88.64	-3.68	-6.72; -0.53	Decreasing
POLIO	North	88.16	61.27	74.17	-4.43	-8.13; -0.58	Decreasing
	Northeast	100.44	66.69	82.19	-5.16	-8.99; -1.17	Decreasing
	Southeast	100.52	70.67	85.72	-4.65	-7.65; -1.55	Decreasing
	South	95.57	79.23	88.18	-1.72	-3.20; -0.21	Decreasing
	Midwest	97.88	73.50	86.57	-4.10	-5.34; -2.84	Decreasing
	Total	98.29	69.91	83.89	-4.68	-7.73; -1.54	Decreasing
OHRV	North	83.09	62.65	75.00	-3.75	-7.45; 0.09	Stationary
	Northeast	94.25	66.84	82.64	-4.44	-7.92; -0.82	Decreasing
	Southeast	98.52	70.83	86.73	-4.86	-7.58; -2.07	Decreasing
	South	98.23	80.53	90.11	-2.34	-4.16; -0.47	Decreasing
	Midwest	95.27	74.87	87.52	-3.78	-5.33; -2.20	Decreasing
	Total	95.35	70.46	84.84	-4.29	-6.95; -1.55	Decreasing
MENINGO-C	North	87.19	64.90	77.40	-3.65	-6.35; -0.86	Decreasing
	Northeast	97.40	67.29	84.39	-5.34	-8.88; -1.66	Decreasing
	Southeast	100.81	70.82	87.22	-5.14	-7.62; -2.60	Decreasing
	South	101.48	80.63	91.38	-2.87	-4.66; -1.05	Decreasing
	Midwest	97.35	75.35	89.19	-3.99	-5.45; -2.51	Decreasing
	Total	98.19	70.89	86.09	-2.51	-7.24; -2.08	Decreasing
YF	North	75.34	51.01	65.84	-6.05	-9.56; -2.40	Decreasing
	Northeast	38.90	43.24	36.36	0.92	-3.37; 5.39	Stationary
	Southeast	31.17	63.65	52.56	15.18	1.50; 30.71	Increasing
	South	58.05	69.38	64.75	4.59	0.37; 8.98	Increasing
	Midwest	87.17	66.81	79.50	-5.26	-6.76; -3.73	Decreasing
	Total	46.31	57.66	53.59	4.72	-0.34; 10.04	Stationary

APC: Annual Percentage Change; CI: Confidence Interval; BCG: Bacillus Calmette and Guérin; HEPB: Hepatitis B; PENTA: Pentavalent; PNEUMO-10: 10-valent pneumococcal; POLIO: Poliomyelitis; OHRV: Oral Human Rotavirus Vaccine; MENINGO-C: Conjugate Meningococcal C; YF: Yellow Fever.

**Table 3:** Time series corresponding to the vaccination coverage rate by immunobiological agent (%) in children under two years old. Brazil, Regions, from 2015 to 2021.

	Region	Rate 2015	Rate 2021	Rate Mean	APC	95%CI	Trend
SCR-D1	North	85.60	67.80	78.84	-3.05	-7.53; 1.66	Stationary
	Northeast	95.31	69.16	87.99	-4.74	-9.17; -0.09	Decreasing
	Southeast	99.92	73.93	90.18	-4.35	-7.30; -1.31	Decreasing
	South	96.12	82.73	89.59	-1.83	-3.38; -0.25	Decreasing
	Midwest	93.73	78.56	88.02	-3.13	-5.02; -1.20	Decreasing
	Total	96.07	73.49	88.09	-3.75	-6.82; -0.57	Decreasing
SCR-D2	North	62.76	34.76	61.75	-7.94	-19.14; 4.81	Stationary
	Northeast	80.35	45.54	64.89	-6.72	-13.90; 1.05	Stationary
	Southeast	86.97	58.55	75.83	-5.62	-10.02; -1.01	Decreasing
	South	76.54	62.76	80.84	-2.47	-7.89; 3.27	Stationary
	Midwest	72.57	44.71	74.31	-6.68	-15.14; 2.62	Stationary
	Total	79.94	51.62	71.80	-6.11	-12.79; 1.07	Stationary
SCRV	North	58.01	1.64	50.48	-32.85	-59.55; 11.46	Stationary
	Northeast	77.05	4.88	27.71	-43.67	-53.14; -32.29	Decreasing
	Southeast	86.98	4.73	34.09	-42.31	-48.33; -35.59	Decreasing
	South	70.69	13.46	64.57	-17.78	-35.37; 4.60	Stationary
	Midwest	68.23	5.61	61.49	-24.50	-46.17; 5.91	Stationary
	Total	77.37	5.74	40.83	-30.18	-40.11; -18.60	Decreasing
HEPA	North	86.67	57.23	71.02	-3.78	-9.04; 1.77	Stationary
	Northeast	94.35	60.93	76.56	-4.38	-9.22; 0.71	Stationary
	Southeast	101.02	69.88	81.45	-2.37	-7.19; 2.70	Stationary
	South	101.63	76.46	85.90	-1.44	-5.52; 2.82	Stationary
	Midwest	93.56	70.09	82.22	-3.06	-6.23; 0.22	Stationary
	Total	97.07	66.87	79.61	-2.62	-7.02; 2.00	Stationary
PNEUMO-B1	North	72.13	60.69	70.61	-2.03	-6.51; 2.66	Stationary
	Northeast	86.40	63.12	76.90	-4.48	-8.21; -0.59	Decreasing
	Southeast	92.90	64.59	79.85	-5.01	-8.09; -1.83	Decreasing
	South	92.51	72.05	83.35	-3.33	-6.07; -0.52	Decreasing
	Midwest	87.73	71.85	82.42	-2.49	-5.10; 0.20	Stationary
	Total	88.35	65.36	78.72	-4.02	-7.06; -0.88	Decreasing
MENINGO-B1	North	72.06	62.51	73.28	-2.49	-5.81; 0.95	Stationary
	Northeast	86.25	64.27	79.96	-4.51	-7.37; -1.56	Decreasing
	Southeast	93.07	68.11	81.99	-4.37	-6.87; -1.80	Decreasing
	South	90.23	76.71	87.16	-2.68	-5.20; -0.10	Decreasing
	Midwest	85.06	72.91	85.34	-3.94	-6.80; -0.98	Decreasing
	Total	87.85	67.98	81.46	-3.79	-6.11; -1.42	Decreasing
DTP-B1	North	71.62	52.87	60.42	-1.80	-4.11; 0.57	Stationary
	Northeast	86.18	57.23	68.63	-3.96	-6.30; -1.55	Decreasing
	Southeast	90.23	65.71	72.28	-1.83	-6.00; 2.53	Stationary
	South	84.41	73.43	75.07	1.53	-1.70; 4.87	Stationary
	Midwest	83.68	65.93	71.42	-3.48	-8.37; 1.68	Stationary
	Total	85.78	62.97	70.29	-1.95	-5.05; 1.24	Stationary
POLIO-B1	North	71.68	51.93	60.75	-2.72	-6.89; 1.65	Stationary
	Northeast	86.53	52.77	68.85	-5.65	-9.07; -2.11	Decreasing
	Southeast	88.50	63.01	75.66	-4.18	-6.08; -2.25	Decreasing
	South	80.10	69.52	78.74	-1.39	-3.18; 0.43	Stationary
	Midwest	83.42	63.97	76.09	-3.22	-5.01; -1.39	Decreasing
	Total	84.52	59.86	72.60	-3.95	-6.24; -1.60	Decreasing

APC: Annual Percentage Change; CI: Confidence Interval; SCR-D1: Triple Viral Dose 1; SCR-D2: Triple Viral Dose 2; SCRV: Tetra Viral; HEPA: Hepatitis A; PNEUMO-B1: Pneumococcal Booster 1; MENINGO-B1: Meningococcal Booster 1; DTP-R1: Triple Bacterial Booster 1; POLIO-R1: Poliomyelitis Booster 1.

Table 4 presents the time series corresponding to the vaccination abandonment rate by multidose immunobiological agent (%) among children under two years old in Brazil, both in the entire country and according to its regions between 2015 and 2016. In the country, a decreasing trend was identified in the abandonment rates for the POLIO and OHRV vaccines, stationary for the PENTA, PNEUMO-10 and triple viral (SCR) vaccines, and increasing for MENINGO-C. The increasing trends in the abandonment rate in the regions of the following immunobiologicals stand out: BCG in the North, Northeast and Midwest; and MENINGO-C in the North and Northeast. In addition to that, many immunobiological agents maintained the stationary trend of this rate in the regions throughout the years under study.

**Table 4:** Time series corresponding to the vaccination abandonment rate by immunobiological agent (%) in children under two years old. Brazil, Regions, from 2015 to 2021.

	Region	Rate 2015	Rate 2021	Rate Mean	APC	95%CI	Trend
PENTA	North	14.87	19.10	19.55	9.11	2.38; 16.28	Increasing
	Northeast	9.31	7.62	11.80	8.01	0.09; 16.57	Increasing
	Southeast	5.25	4.01	6.55	-2.76	-18.72; 6.35	Stationary
	South	6.83	3.80	7.13	3.56	-6.33; 14.49	Stationary
	Midwest	6.73	6.69	9.16	11.43	0.79; 23.19	Increasing
	Total	7.75	6.91	9.73	5.93	-3.21; 15.92	Stationary
PNEUMO-10	North	12.95	8.52	7.48	1.08	-10.21; 13.80	Stationary
	Northeast	8.40	1.84	3.37	-18.59	-26.39; -9.98	Decreasing
	Southeast	4.18	1.32	1.79	-6.77	-39.84; 44.48	Stationary
	South	6.34	1.27	2.05	-16.05	-34.55; 7.67	Stationary
	Midwest	7.27	1.69	2.60	-9.40	-25.84; 10.68	Stationary
	Total	6.81	2.27	2.93	-10.24	-23.15; 4.83	Stationary
POLIO	North	99.70	18.91	30.83	-17.44	-36.61; 7.52	Stationary
	Northeast	99.06	8.43	24.07	-25.94	-44.17; -1.77	Decreasing
	Southeast	96.83	4.59	19.75	-34.58	-53.58; -7.79	Decreasing
	South	99.59	4.96	19.90	-33.90	-55.84; -1.07	Decreasing
	Midwest	99.73	6.99	22.08	-29.47	-49.46; -1.58	Decreasing
	Total	98.83	7.52	22.43	-28.11	-47.51; -1.54	Decreasing
OHRV	North	9.69	8.62	8.31	9.50	-4.50; 25.56	Stationary
	Northeast	10.20	2.61	6.40	-20.00	-26.97; -12.37	Decreasing
	Southeast	6.48	1.88	3.55	-14.84	-29.47; 2.82	Stationary
	South	5.18	1.70	2.94	-11.63	-23.84; 2.55	Stationary
	Midwest	4.62	2.40	3.72	-6.57	-20.49; 9.79	Stationary
	Total	7.55	2.81	4.77	-12.36	-21.92; -1.63	Decreasing
MENINGO-C	North	7.87	10.67	9.05	10.37	0.35; 21.40	Increasing
	Northeast	3.17	6.06	5.13	14.59	5.01; 25.04	Increasing
	Southeast	0.48	2.70	1.56	7.75	-57.53; 173.39	Stationary
	South	1.96	2.99	2.10	17.52	-15.95; 64.31	Stationary
	Midwest	2.10	4.08	2.77	27.83	-0.86; 64.82	Stationary
	Total	2.32	4.68	3.53	18.33	1.35; 38.16	Increasing
SCR	North	32.45	48.82	23.13	8.72	-11.84; 34.08	Stationary
	Northeast	20.09	34.26	27.40	2.13	-7.71; 13.01	Stationary
	Southeast	14.66	21.02	16.43	5.09	-6.79; 18.49	Stationary
	South	27.73	24.40	10.81	4.53	-29.53; 55.04	Stationary
	Midwest	29.04	43.19	17.05	10.55	-19.39; 51.61	Stationary
	Total	20.77	29.89	19.48	4.34	-11.55; 23.08	Stationary

APC: Annual Percentage Change; CI: Confidence Interval; PENTA: Pentavalent; PNEUMO-10: 10-valent pneumococcal; POLIO: Poliomyelitis; OHRV: Oral Human Rotavirus Vaccine; MENINGO-C: Conjugate Meningococcal C; SCR: Triple Viral.

## DISCUSSION

The study results pointed out important aspects related to the vaccination of children under two years old in Brazil, highlighting the decreasing VC rate and the stationary abandonment rate during the period analyzed. This indicates a considerable deterioration in recent years in the vaccination status of this population group in the country, as immunobiologicals in the basic vaccination schedule such as SCRV (77.37%), SCR-D2 (79.94%) and YF (46.31%) did not achieve desirable coverage rates as early as 2015. Furthermore, there has been a significant drop in VC since then, falling short of what is recommended by the Brazilian Ministry of Health, with most vaccines presenting VC values below 70%.

In the population group analyzed, vaccination coverage is considered adequate when it reaches values equal to or greater than: 90.00% for BCG and HRV; and 95.00% for the POLIO, PENTA, HEPB, MENINGO-C, PNEUMO-10, YF and HEPA vaccines.^17,20,21^ Therefore, from the results it can be seen that none of the vaccines reached an adequate VC in 2021 when we consider the total. Regarding the regions, the results indicate that, in general, the lowest VC values are in the North and Northeast regions; this is possibly due to difficulties accessing vaccination services, to availability of products and, not least, to geographic access difficulties in both regions.^4^

It is evident that, throughout its history, the Brazilian PNI has become increasingly complex with the inclusion of new vaccines in the basic schedule routinely offered in health services, which resulted in a favorable change in the epidemiological scenario of vaccine-preventable childhood diseases in the country.^2,5^ However, in view of these results, the number of children that have not completed the vaccination schedule is still significant, even though these vaccines are available in the Brazilian health system free of charge. This is because vaccination success goes beyond availability and supply of vaccines, being influenced by lack of confidence and acceptance of the vaccines, by the services operating at inappropriate times for the population and by fear of adverse events after vaccination, among others.^5^

A study that analyzed VC for HEPA, CVS and Varicella among children aged 15 months old in the Brazilian state of Minas Gerais also showed low VC in 2020.^13^ In São Paulo, another Brazilian state, a number of researchers investigated VC specifically for measles, an infectious disease included in the country's basic vaccination schedule, and identified low VC between 2015 and 2020, with a greater reduction for the second dose of the vaccine against the disease.^14^ Also in this regard, a research study that evaluated VC for agents that cause respiratory tract infections and the prevalence of childhood hospitalizations for these conditions in Brazil from 2015 to 2020 also found low VC and a significant increase in hospitalizations due to measles during the same period.^22^

Regarding the trend of VC rates by each immunobiological specifically, a decreasing or stationary trend was observed in all regions, with the exception of the YF vaccine, which was increasing in the South and Southeast regions. This points to certain risk for the reintroduction or resurgence of controlled or eradicated diseases in the country, a phenomenon observed worldwide, especially since 2016.^6^ In this way, children under the age of two become more vulnerable to contracting serious and life-threatening diseases such as severe forms of tuberculosis, hepatitis b, diphtheria, tetanus, pertussis, poliomyelitis, rotavirus diarrhea, pneumonia, meningitis, yellow fever, measles, mumps, pertussis, hepatitis a, varicella and rubella. Because of this scenario, the emergence of diseases that had already been eliminated has been observed, such as measles, eradicated in the country in 2016.

In this sense, according to the epidemiological bulletin of the Brazilian Ministry of Health, the last measles cases in the country were reported in 2015 and, in 2016, the country received a certification corresponding to elimination of the virus. Consequently, in 2016 and 2017, no measles cases were confirmed in the country. However, in 2018, 10,346 cases of the disease were reported. In 2019, after a year of free circulation of the virus and the confirmation of 20,901 cases of the disease, Brazil lost the "measles-free country" certification.^23^ This scenario put health authorities in Brazil and the Americas on alert, as other diseases had already been controlled or even eradicated, as was the case with smallpox, poliomyelitis, rubella and congenital rubella,^5,24,25^ making it necessary to intensify actions aimed at achieving the VC rate for all immunobiological agents in the basic vaccination schedule.

Over the years, many studies have shown the positive impact of vaccination on hospital admissions and infant mortality. Thus, it is estimated that, worldwide, approximately 2.5 million deaths are prevented every year thanks to compliance with the basic vaccination schedule for children under five years old. However, at least 20.00% of the children born each year do not receive the benefits of vaccination and are exposed to the risks of becoming ill and dying before the age of five.^26^ A study that described the impact of introducing the SCRV vaccine in the Brazilian PNI in 2013 for 15-month-old children showed that vaccination significantly reduced the mortality and hospitalization rates associated with varicella in Brazil. In addition, the country presented a 57.00% drop in the mortality rate among infants and a 49.00% reduction in children aged from one to four years old. In terms of hospitalizations, the reductions were 40.00 and 47.00%, respectively.^27^

However, when comparing the findings of this research with the global infant VC rates, according to a report published in 2022 by the WHO in partnership with UNICEF, it is observed that the global VC dropped from 86.00% in 2019 to 81.00% in 2021; in other words, the number of non-vaccinated children increased by 5 million from 2019 to 2021. Thus, it is estimated that, worldwide, 25 million children under one year old did not receive the vaccines in the basic schedule, the highest number since 2009.^15^

It is also noteworthy that the 62.30% VC among children under two years of age in Brazil in 2021 was below the global rate (81.00%). This highlights the weakness of the PNI in the country which, despite currently offering the largest number of vaccines free of charge (17 immunobiologicals for children under two years of age in the basic calendar), is among the ten worst nations in the world in terms of vaccination.^3^

Another result that was highlighted in the current study refers to the vaccine abandonment rate among children under two years old in Brazil, which proved to be stationary and growing for most immunobiologicals during the period analyzed, especially with the increasing BCG abandonment rate in the North, Northeast and Midwest regions, and MENINGO-C also in the North and Northeast regions. Also regarding this aspect, there was an increase in the overall abandonment rate during the last three years analyzed, from 6.40% in 2019 to 9.00% in 2021. In this sense, a recent study that analyzed the MMR vaccine abandonment rate in Brazil according to regions of the country, from 2014 to 2021, corroborated the results found in the current research since, during the period investigated, the abandonment rate varied among the Federation Units that make up the Brazilian regions, ranging from 22.20% in 2014 to 37.40% in 2021.^7^ Therefore, the abandonment rate is considered average, and investments are required to reduce it in the country.^6^

Some studies have pointed out the factors that contribute to the decrease in VC and the increase in the abandonment rate, such as fear of a post-vaccination reaction, lack of knowledge on the part of parents about the importance of vaccination and about the severity of vaccine-preventable diseases, the socioeconomic conditions and geographic characteristics of the population, the working conditions of the nursing professionals who work in vaccination rooms, dissemination of fake news about vaccines, an increase in anti-vaccination movements and false contraindications.^9,28^

It is evident that the advent of the infection caused by SARS-CoV-2, considered a pandemic by the WHO in March 2020,^29^ also emerged as an important factor that negatively influenced the VC and vaccine abandonment rates among children under two years old in Brazil and in the world. In 2020 and 2021, Brazil faced the worst moment of the COVID-19 pandemic, which strongly modified how health services were resorted to, in which face-to-face attendance dropped dramatically even for childhood vaccinations, due to social distancing measures implemented in order to reduce transmission of the virus, in addition to the parents’ fear of contracting COVID-19 when taking their children to be vaccinated.^30^

A study carried out in Jordan corroborates these findings, as a decline in the VC of Jordanian children was identified during the COVID-19 pandemic in 2020, highlighting the importance of formulating future strategies to promote vaccination and avoid setbacks in the vaccination rates during new waves of the COVID-19 or other pandemics. The researchers also signal the need to improve the health services, alleviate the caregivers’ concerns about the coronavirus contamination risk and organize vaccination campaigns outside health centers.^11^

Regarding strategies to improve the vaccination rates, in the international scenario, research studies carried out in Mexico, Colombia, Paraguay and Venezuela point to initiatives to reduce vaccine abandonment and achieve VC in children. Among them, an active search for newborns to adhere to the vaccination program, as well as their follow-up until conclusion of the recommended schedule, taking advantage of all contacts with health services to vaccinate them and also discussing false contraindications for children, as the mothers’ knowledge about vaccines was pointed out as a decisive aspect for acceptance of the immunobiologicals.^8-10^

It is also noteworthy that the results indicate that there are different behaviors between vaccines that should be carried out at the same time, such as the first DTP booster and the poliomyelitis booster, recommended at 15 months of age. This distinction may be due to unavailability of certain immunobiologicals in the services or to specific vaccine hesitancy.^12^

Thus, the importance of monitoring the VC and abandonment rates in all Brazilian regions is reinforced, in order to support the development of strategies to achieve the recommended VC and reduce vaccine abandonment, protecting children against countless serious preventable diseases. Health professionals need to engage as health educators to discuss with the population the role of immunobiologicals in disease prevention, as well as to update the vaccination schedule whenever appropriate.

Access to secondary databases as an information source stands out as a study limitation since, despite being an official source from the Brazilian Ministry of Health, it may present inconsistencies in relation to data feeding and updating. Some atypical extreme values are common in municipalities with a small population (less than one hundred children), where the quality of state data may be compromised, as any variation in the birth rate and/or registration of the dose applied alters the behavior of the target indicator performance, with rates higher than 100.00%.^4^ Therefore, such overestimated or underestimated values at the levels at which these indices are found are questionable and it was not possible to include them in the context of adequate VC; therefore, despite being a good indicator to suggest "population immunity", they should be viewed with caution.

In conclusion, the study results show a worrisome scenario in Brazil, with a stationary or decreasing vaccination coverage rate trend in children under two years old for all the immunobiologicals in all Brazilian regions, with the exception of YF in the South and Southeast regions. There was an increase in the abandonment rate trend for the Meningococcal C vaccine in the country. In relation to the regions, there was an increase in the abandonment rate for BCG in the North, Northeast and Midwest and for Meningococcal C in the North and Northeast. Finally, new studies must be carried out in order to understand the individual and subjective aspects involved in children's vaccination behavior, including their parents’/guardians’ perspective, in order to target and devise strategies for monitoring and increasing the VC rate and reducing the abandonment rate.

## Data Availability

The database that originated the article is available with the corresponding author.
